# SASI-Seq: sample assurance Spike-Ins, and highly differentiating 384 barcoding for Illumina sequencing

**DOI:** 10.1186/1471-2164-15-110

**Published:** 2014-02-07

**Authors:** Michael A Quail, Miriam Smith, David Jackson, Steven Leonard, Thomas Skelly, Harold P Swerdlow, Yong Gu, Peter Ellis

**Affiliations:** Wellcome Trust Sanger Institute, Hinxton, CB10 1SA Cambs, UK; Frederick National Laboratory for Cancer Research, Leidos Biomedical Research, Bldg. 427, 21702-1201 Frederick, MD USA

**Keywords:** Next-generation sequencing, Indexing, Barcode, Illumina, Sample assurance, Spike-in, Contamination, Sample identity

## Abstract

**Background:**

A minor but significant fraction of samples subjected to next-generation sequencing methods are either mixed-up or cross-contaminated. These events can lead to false or inconclusive results. We have therefore developed SASI-Seq; a process whereby a set of uniquely barcoded DNA fragments are added to samples destined for sequencing. From the final sequencing data, one can verify that all the reads derive from the original sample(s) and not from contaminants or other samples.

**Results:**

By adding a mixture of three uniquely barcoded amplicons, of different sizes spanning the range of insert sizes one would normally use for Illumina sequencing, at a spike-in level of approximately 0.1%, we demonstrate that these fragments remain intimately associated with the sample. They can be detected following even the tightest size selection regimes or exome enrichment and can report the occurrence of sample mix-ups and cross-contamination.

As a consequence of this work, we have designed a set of 384 eleven-base Illumina barcode sequences that are at least 5 changes apart from each other, allowing for single-error correction and very low levels of barcode misallocation due to sequencing error.

**Conclusion:**

SASI-Seq is a simple, inexpensive and flexible tool that enables sample assurance, allows deconvolution of sample mix-ups and reports levels of cross-contamination between samples throughout NGS workflows.

**Electronic supplementary material:**

The online version of this article (doi:10.1186/1471-2164-15-110) contains supplementary material, which is available to authorized users.

## Background

As NGS matures and sequence yields increase, the scale of sequencing projects being undertaken is ever increasing. There are now many sequencing projects tackling thousands, or tens of thousands of samples; e.g., the UK10K project (http://www.uk10k.org) and the malaria genome consortium [1]. Large sample numbers from both case and control sets are commonly being sequenced in order to detect rare alleles that are associated with disease. Sample contamination and mix-ups are a serious problem, and can interfere with the sensitive statistical methods being used to determine such causal variants [2–7]. Whilst laboratories can implement elaborate tracking procedures involving barcoding and automated handling, sample swaps, plate swaps, and cross-contamination can still occur [8, 9]. Recent analyses using *cox*I phylogenetic relationships suggest that up to 5% error may exist in sequence database entries [10], but do not have the power to determine the cause of that error. In the human genome project, clone identity could be verified by crossmatching *in-silico* digestion patterns of the final sequence against DNA fingerprinting information generated during physical map construction [11]. In the 1000 genome project [12] sample identity was verified by comparison of sequence variation to the Hap Map database information for the corresponding sample and bioinformatics tools were written to assess levels of cross-contamination (e.g. ContEST [13] and subsequently VerifyBAM [7]). These approaches however are expensive, requiring significant work that may preclude their use for larger sequencing projects and fast turn-around clinical sequencing projects. Furthermore, they are sometimes not sensitive enough to unambiguously identify a sample and they report only the bulk properties of a sample and would therefore not be able to report minor cross-contamination events.

**Figure 1:**
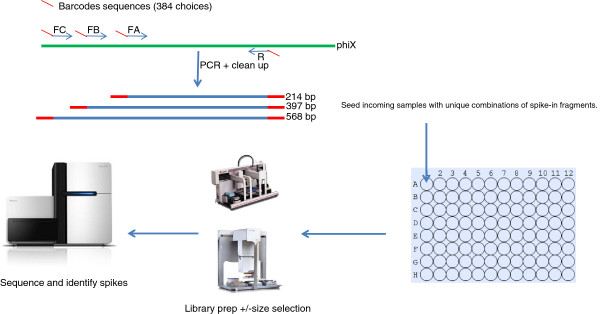
**Diagrammatic representation of the SASI-Seq process.** Amplicons of a reference sequence (here we use PhiX174) are generated with unique barcodes at their 5’ end. Sets of amplicons with different barcodes are added to each sample that is destined for sequencing. The SASI fragments stay with the sample through library prep and can be detected after sequencing. SASI-Seq thus verifies which sample the sequence data originated from. FA, FB and FC represent the forward primers for the 214, 397 and 568 bp SASI fragments respectively, with SASI barcodes at the 5’ end shown here in red. R is the reverse SASI fragment primer also having a SASI barcode at the 5’ end, here coloured in red. Primer sequences are detailed in Methods.

Thus, we have conceived SASI-Seq (**S**ample **A**ssurance **S**pike-**I**n sequencing) whereby uniquely barcoded DNA fragments are spiked into samples at the onset. A given SASI tag will stay intimately associated with a sample as it is processed through library preparation and sequencing set-up (Figure 1). The sequence of that tag will be read at the same time that a sample is sequenced, thus allowing unambiguous identification of a sample by virtue of its reported SASI tag sequence. The spike-in can be done at low levels that would nonetheless generate a large enough number of reads to enable identification of minor contaminants. The idea of spiked-in fragments is not new; ERCC RNA spike-ins [14] are routinely used to normalise RNA expression levels between different experiments, combinations of primer pairs specifying control fragments of defined length have been advocated for genotyping studies [15] and Illumina include optional spike-in fragments, to diagnose the efficiency of library preparation steps, within their TruSeq kits.

The present method, however, has much wider utility, providing assurance that a sequence has come from the correct sample. Without such assurance, sample swaps and cross-contamination often go unnoticed, resulting in erroneous or confusing results, both of which could be disastrous for clinical sequencing applications.

With the introduction of massively parallel next-generation sequencing technologies came the realisation that a single sequencing run often yielded too many reads, particularly for smaller genomes and amplicons. Methods were developed to multiplex samples, involving the addition of a different unique short barcode sequence to each sample during library preparation. Subsequently, they could be mixed, sequenced together and the reads correctly attributed to the appropriate sample by binning reads containing the same barcode sequence. This practice was first reported for Roche 454 sequencing [16, 17], and soon after for the Illumina platform [18]. As sequencing yields have risen higher, the degree of multiplexing has also risen, with Kozarewa and Turner (2011) reporting a set of 96 barcodes [19], Caporaso *et al*., (2012) describing a set of 2167 barcodes [20] and Costea *et al*., (2013) developing the software tool TagGD that can design up to 20,000-plex barcode sets [21], for use in Illumina sequencing. These Illumina barcodes were designed using a Hamming script [22, 23] with an edit distance of 4 (i.e. such that no barcode sequence was less than 4 changes away from its closest sequence match). In theory, this allows single nucleotide errors to be corrected, and two errors to be detected, without reporting the wrong barcode. Here we report a set of 384 barcodes with an edit distance of 5 between any two members, allowing single base error correction, three base error detection and requiring at least 4 sequencing errors in the barcode read before being mistaken for an alternative barcode.

## Results

### SASI fragment design

For the SASI-Seq approach to work, a fragment or set of fragments was required that would be inexpensive, easily identified and resistant to degradation and loss during Illumina library preparation. One of the most common variable steps in the library preparation process is size selection [24], which can yield very tight (+/- 10 bp) or very broad (+/- 400 bp, or greater) fragment size ranges. In order to prevent the SASI fragments being lost during size selection, we therefore envisioned a set of three fragments of different sizes, approx. 200 bp, 400 bp and 600 bp that would be evenly spaced within the range of fragment size distributions commonly used for Illumina sequencing.

The viral genome PhiX 174 is easily one of the most commonly sequenced genomes, as it is often used as an internal control during Illumina sequencing [25, 26]. As such, it likely has a perfect reference and bioinformatics pipelines have been written to remove PhiX reads from Illumina datasets. We therefore designed our spike-in fragments to represent discrete segments of the PhiX genome around a common core. To do this, we used the program Oligo 6 [27] to design a set of primers against the NC_001422.1 Genbank reference sequence, that gave three fragments of approximately 200 bp, 400 bp and 600 bp from a common reverse primer and that had roughly equal Tm and priming efficiencies. The best primer pairs had forward primers at positions 926, 743 and 571 and a reverse primer at position 1123 giving amplicons of 214, 397 and 568 bp respectively. In order to add a unique signature to these fragments that could be uniquely associated with a particular sample, we placed a unique sequence barcode from our set of Illumina barcode sequences at the 5’ end of each forward primer [28]. These barcodes were designed using a Hamming script [22, 23] that considers that the major error mode of Illumina sequencing is substitution errors and ensures that no two barcodes are less than 4 base substitutions apart. This enables single error correction i.e. if a barcode sequence gains an error during sequencing it will be one base away from the perfect sequence and can be counted as that original barcode. A barcode sequence has to gain at least three errors before it will be falsely counted as an alternative barcode. With the Illumina error rate less than 1% [29] this should occur at a frequency of less than 1 in 10^6^. For the purposes of both this application and for multiplexing during Illumina sequencing we sought to construct a set of 384 such barcodes that included our previous set of 8mer 96 multiplexing barcodes [19, 30, 31]. To do this we found we needed to expand the barcode word length to be a 9mer, so assigned the 9^th^ base as A in the first 167 barcodes in the set as this is the first base of the Illumina adapter sequence following the run of barcode bases (for 9mer barcode sequences see Additional file 1: Table S1).

### Initial SASI fragment investigation experiments

**Figure 2:**
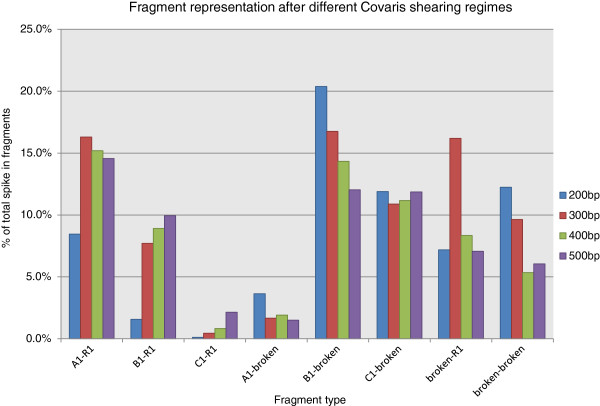
**Percentage of SASI fragments detected after different Covaris shearing regimes.** With all shearing conditions, some intact A, B and C fragments with barcode number 1 (here referred to as A1-R1, B1-R1 or C1-R1) are detected. In general the more intense the shearing regime, the greater number of partial, or broken amplicons (fragments with terminal sequences A1, B1, C1 or R1 at one end and an internal sequence at the other end, here referred to as A1-broken, B1-broken, C1-broken or R1-broken), are detected. The longer amplicons are more susceptible to breakage during shearing than the smallest A1-R1 amplicon (214 bp).

In order for this approach to work it was necessary to demonstrate that the SASI fragments remain within a DNA sample once added and could not be degraded or processed away. For these tests SASI amplicons were generated with barcode tag #1 at both ends, as described in Methods. We sought to determine whether or not the fragments were sheared using typical physical shearing conditions employed during Illumina library construction. 500 ng aliquots of human genomic DNA were spiked with 0.5 ng of SASI fragment mixture and sheared using a Covaris focused acoustic shearing device to produce average fragment sizes of 200 bp, 300 bp, 400 bp and 500 bp respectively. Illumina sequencing libraries were constructed from each sheared DNA sample and with each library receiving a different P7 indexing barcode sequence. The libraries were mixed in equimolar proportions and sequenced on an Illumina MiSeq instrument. From each indexed library we analysed the fraction of reads that shared similarity to the PhiX reference sequence (Figure 2). This clearly demonstrated that the majority of the SASI fragments were broken during shearing, that virtually none of the larger 568 bp amplicon remained, but approximately 10% of detected fragments were intact 214 and 397 bp amplicons.

**Figure 3:**
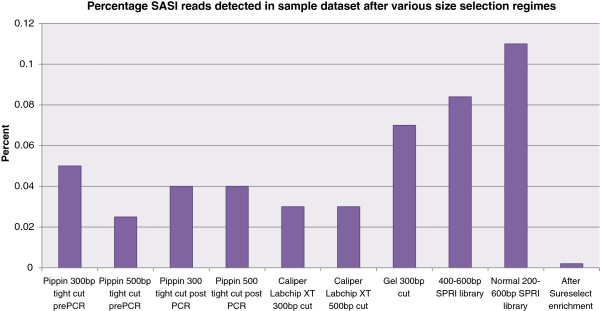
**Percentage of SASI fragments detected after different size selection regimes.** Approximately 0.1% of the sequences obtained from the “normal” 200-600 bp SPRI library were SASI-fragments which corresponds well to the 0.1% initial spike-in dosage. With more stringent size selection, SASI fragments could still be detected, albeit at a lower level.

In order to investigate the effect of different size selection protocols on the levels of detectable SASI fragments, we again took 500 ng aliquots of human genomic DNA spiked with 0.5 ng of SASI fragment mixture, sheared to an average fragment size of 300 bp and made Illumina sequencing libraries using a variety of size selection approaches, before sequencing as a multiplexed pool. We have previously found the Sage Science Pippin Prep gives the tightest distribution of fragment sizes during fractionation [24]. We therefore used the Pippin Prep to separate as tight a size fraction as possible centred around 300 or 500 bp, i.e. approximately halfway between the sizes of the SASI amplicons. We made libraries including this size fractionation step both before and after library PCR. We also made libraries using: the Caliper LabchipXT to size fractionate tight 300 and 500 bp fragments; agarose-gel electrophoresis to size fractionate a tight 300 bp size fraction; AMPure beads to purify >200 bp fragments and 400-600 bp fragments; and Agilent SureSelect custom exome enrichment. The results demonstrated the persistent nature of the SASI fragments in that we were able to detect SASI fragment reads from all the libraries (Figure 3), including after Pippin Prep fractionation (for mapped insert-size distributions, see Additional file 2: Figure S1), and perhaps surprisingly, even after SureSelect target enrichment, albeit at a very low level.

Following the inclusion of a specific probe (10 μM final concentration, for details see Methods) we found that SASI fragments could be reproducibly detected following SureSelect target enrichment with representation after sequencing, close to the spike-in level (results not shown).

### Optimisation of multiplexing barcode sequences

Ideally, sequence multiplexing would be pure in the sense that a single sample would have a unique and exclusive barcode sequence. Also for SASI-Seq to have maximum sensitivity, a single sample would only display the intended barcode sequence(s). However, there are two mechanisms by which background contamination can occur: i) cross-contamination can occur between barcoding oligonucleotides during synthesis and subsequent processing and ii) errors during sequencing can lead to sequence drift such that an alternate barcode sequence is read.

In previous experiments in which samples were deliberately omitted from multiplexed library pools, we noticed that such samples could still be detected at a low level. In order to determine the best processing and purification approach for oligo synthesis, we made a set of libraries using barcoded multiplexing PCR primers that had been purified by HPLC (from Company A) or PAGE purification (Company B), or using IDT TruGrade processing (custom service, Integrated DNA Technologies, Iowa, USA). We deliberately did not open the tubes containing some of the barcode primers, but included those barcodes in the sequence dataset analysis, looking to see what fraction of reads were attributed to those barcodes, although they had not been used (Additional file 3: Table S2). With HPLC or PAGE purification, approximately 0.56% and 0.34% of reads mapped to the missing barcodes. With TruGrade this was dramatically reduced to just 0.03%. The set of barcode sequences used initially was designed to be 4 bases apart and to tolerate one mismatch. In order to investigate the origin of these mis-attributed barcodes, we tabulated the number of perfect matches and the number of matches within one mismatch against each barcode in the 384 set (Additional file 4: Table S3 sheet1). We found that some matches to absent barcode sequences had higher levels of perfect matches (than single-change mismatches) to other barcodes synthesised within the same batch, indicating cross-contamination in the lab or during synthesis. Other mis-attributed barcodes had higher numbers of hits allowing for one mismatch than they did to perfect matches, indicating that those matches were due to sequence drift from other barcodes as a result of sequencing error. We looked at the level of barcode mis-attribution in other runs, two of which are illustrated in Additional file 4: Table S3 as sheets 2 and 3. Whilst in some runs mis-attribution was primarily due to perfect matches indicating lab contamination (Additional file 4: Table S3 sheet2), upto 0.2% mis-attribution was observed due to sequence drift (Additional file 4: Table S3 sheet 3).

In order to make SASI-Seq as sensitive as possible, and sample multiplexing as distinct as possible, we sought to reduce this background level of barcode mis-attribution by redesigning our 384 plex barcode set such that they were at least 5 bases different from the closest other barcode sequence. When using single error correction, this would tolerate 3 sequencing errors, since at least 4 sequencing errors would be required to potentially convert each to within one base of an alternative barcode. This required increasing the barcode length to 11 bases, the sequences of which are given in Additional file 5: Table S4.

We ordered Illumina PCR multiplexing oligos with these 384 different 11-mer barcode sequences from IDT to TruGrade purity in four 96 well plates, and validated that purity by checking for the presence of unexpected barcodes. Briefly we amplified an Illumina adapter ligated fragment library of the *S. aureus* TW20 strain, in the presence of each of the 384 barcoded primers, in four 96 well plates. After PCR we made two multiplexed library pools, one containing an equal volume of all odd number barcoded libraries and the other with an equal volume of all the even number barcoded libraries. We used an 8-channel pipette for this purpose, so that we could pipette whole columns without error. Each pool was purified, quantified and run on an Illumina MiSeq to determine the frequency of each barcode (Additional file 6: Table S5). The incidence of mis-attribution in each experiment was less than 0.005%, of which 75% and 83% respectively were perfect matches, demonstrating the highly discriminatory nature of these barcodes which would be a prerequisite for sensitive cross-contamination detection using SASI-Seq.

### Sample assurance using SASI-Seq

**Figure 4:**
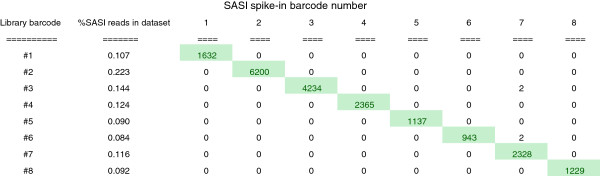
**Demonstration of the utility of SASI-Seq for sample assurance.**

To test the performance of SASI-Seq, we prepared a set of 96 multiplexed libraries from samples that had been spiked with 0.1% SASI fragments containing a unique 11-mer barcode at one end. The reads from each library were segregated according to the barcode sequence and each library dataset mined for reads originating from the SASI spike-in fragments. The results are best visualised as a tabulated matrix of sequencing barcode versus spike-in barcode for each library (e.g. (Figure 4), full results in Additional file 7: Table S6). The number of SASI specific reads varies between samples, but for each their representation roughly approximates the 0.1% spike-in level. Variation is probably a result of a number of factors including variation in the number of reads for each Illumina barcode data set, accuracy of quantification of both DNA sample and SASI fragments as well as pipetting accuracy at low volumes. In separate experiments (not shown) different relative levels of sequencing barcodes and SASI fragments were observed indicating that some barcodes/fragments are not outperforming others as has been observed with some “in-line” barcoding sets (e.g. [32]).

For the most part, the only SASI fragments detected in each library dataset were those that were expected. There were, however, a small number of hits to other SASI barcode sequences in some of the libraries. On analysis, these were found to be perfect matches indicating cross-contamination during processing rather than sequence error resulting in barcode cross-talk.

### Detecting sample swaps and cross-contamination using SASI-Seq

**Figure 5:**
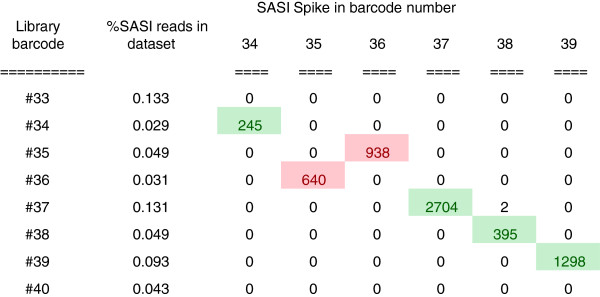
**Demonstration of the utility of SASI-Seq for detecting sample swaps.**

To demonstrate that we could reliably use SASI-Seq to detect sample swaps and cross-contamination events we deliberately mixed samples with known spike-ins. Sample swaps could be identified quite readily, an example of this is shown in (Figure 5), in which two consecutive samples were purposely transposed.

**Figure 6:**
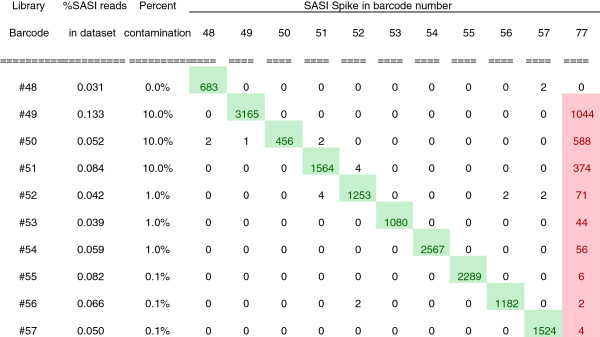
**Demonstration of the utility of SASI-Seq for detecting cross-contamination.**

We next tested the power by which we could detect cross-contamination by deliberately mixing in samples containing other spike-in barcodes at known levels. Specifically, samples in triplicate containing 0.1% uniquely tagged spike-ins were deliberately cross-contaminated by adding another sample, containing 0.1% spike-ins with SASI barcode #77, to 10%, 1% and 0.1% relative to the concentration of the original sample. At the 0.1% level of overall spike-in, cross-contamination down to 1% could be reliably detected above the background contamination and sample-to-sample SASI read variation, within the experiment (Figure 6). The low level of background contamination seen in this experiment was probably a result of small splashover events during library preparation since the contaminants had perfect matches and no such contamination was observed when individual libraries were remade manually (results not shown).

### Universal SASI-Seq

In order to make the SASI fragment design applicable to Nextera library preparation and PCR-based enrichment approaches, we added the sequences TCGTCGGCAGCGTCAGATGTGTATAAGAGACAG and GTCTCGTGGGCTCGGAGATGTGTATAAGAGACAG, that are normally introduced via the Nextera reaction [33], to the respective 5’ ends of forward and reverse primers used in SASI fragment generation. This enabled the SASI fragments to be amplified using the standard Nextera PCR primers, or using primers with these sequences that could easily be included in any PCR enrichment panel (results not shown).

### SNP calling

Since this approach involves adding foreign DNA to samples under study we had a slight concern that SASI fragment sequence may contaminate usable sequence data and interfere with subsequent analysis, leading to false SNP calling and elevated false positive rates. To examine this possibility we sequenced the genome of Staphylococcus aureus TW20, for which we had a complete genome sequence [34], both with and without the inclusion of SASI fragments. Variant analysis of the resulting datasets showed that neither dataset had any variants compared to the reference thus providing assurance that SASI fragments do not lead to false SNP calls.

## Discussion

Mistakes resulting in sample swaps and cross-contamination can, and undoubtedly do, happen in the laboratory, but no one knows with certainty the true frequency of such events. Due to the rapid uptake and power of NGS, hundreds of thousands of genomes are now being sequenced per annum and that number is increasing. Such throughput can only put strain on the upstream pipelines that feed the sequencing instruments, potentially resulting in more sample swaps and cross-contamination events that could lead to false diagnosis, erroneous conclusions or confuse statistical analyses searching for causative mutations. Moreover, such events are often costly, as investigations to determine the cause of the mistake and determine the true identity of samples can be very time consuming. Here we have demonstrated a simple, inexpensive and flexible method for sample assurance whereby barcoded amplicons are spiked into samples as close as possible to the source and stay with that sample through the sequencing process. This technique has the ability to provide peace of mind by reassuring the user that the obtained sequence is from the desired sample.

Theoretically, there are no real restrictions on the sequence of the amplicons used for this approach. We chose here to use amplicons from a region of the PhiX 174 genome as we would be unlikely to sequence such a genome in a real experiment. Therefore, there is little chance of confusing SASI reads with those of the sample. In order to retain flexibility we utilise a set of three fragments that span the range of fragment sizes commonly used in Illumina sequencing and demonstrate that those fragments persist even after very tight size selection. If one had a standardised protocol where all libraries produced had the same insert size range, it may be possible to use just one such SASI fragment. Since virtually all of the larger 568 bp fragment is fragmented during shearing (Figure 2) it could be argued to be superfluous, however we retain it to allow for the eventually whereby larger size selected fragment libraries are prepared.

For probe-based target enrichment studies, we had envisaged incorporation of a specific capture probe for the region of the PhiX genome covered by the SASI fragments. This approach appeared to work well in that SASI fragment representation after selection was found to be close to input levels. It was a surprise to us that the SASI fragments could be reproducibly detected following Agilent SureSelect exome target enrichment without specific probes being present, and illustrates the “leaky” nature of the solution hybrid capture technique. Whilst not absolutely necessary we would recommend supplementation of the probe capture set with a SASI specific probe to ensure reliability.

Similarly, the method is flexible enough to accommodate other enrichment approaches, provided the SASI fragments are included in the experimental design. In particular for PCR-based enrichment approaches, the SASI fragment design would have to be modified slightly to include sequences at the termini from which the SASI fragments could be amplified, if primers annealing to those regions were to be included in primer panels. Extra flanking sequence is also required for samples bound for Nextera library preparation, in order that the termini of the SASI fragments are appended with the sequences normally added by the transposase enzyme. Adding these sequences enables the fragments to be amplified in the subsequent PCR step. Since this sequence could be used for amplification in PCR-based enrichment approaches, addition of this sequence at the termini of the SASI fragment oligos would be recommended in order to provide sets of fragments with universal application.

Almost by definition, Next-Generation Sequencing is “massively parallel,” generating many millions of individual sequence reads; therefore, there is considerable scope for cross-contamination to be detected. There are several means by which contamination can occur, including not just physical contamination of one DNA sample with another, but by cross-contamination of barcoding oligonucleotides during manufacturers’ processing, or in one’s own laboratory, and by misattribution following sequencing error. Here we have addressed two of the causes by which such contamination can occur. Notably we have increased the edit distance on our Illumina sample barcoding sequences to five, and identified optimal barcoding oligonucleotide purification conditions.

Since we started barcoding libraries in 2009 we have surveyed oligos at different purities from a number of manufacturers with similar results to those presented here. Manufacturers’ literature suggest that HPLC should give >85% purity of full length oligonucleotide and PAGE >90% purity but no specifications or guarantees are given in regard of cross-contamination by other oligonucleotides. Ourselves, and collaborating NGS practitioners, have noted that such cross-contamination is higher following HPLC purification so we have until now ordered barcoding oligonucleotides with PAGE purification even though it tends to give lower overall yields than if HPLC purification is specified. Cross-contamination presumably occurs due to use of common labware in the oligonucleotide synthesis facility. IDT's TruGrade manufacturing utilizes manual and automated processing workflows to minimize the chance of oligo-to-oligo interaction throughout processing.

As a result of this investigation we list a set of 384 highly discriminating barcodes that can have a background rate of false attribution of less than 0.005%. Aside from its application in sample assurance SASI-Seq could be used for barcoding oligo QC. If one had a validated set of samples containing previously determined SASI barcodes, one could amplify that set with each new batch of barcoding oligos and any irregularities in indexing barcode identity would be reported by the SASI barcodes detected. Once the barcoding oligo set and the investigator’s library process have been certified “clean”, SASI-Seq could also be used to measure sample-to-sample or run-to-run contamination in next-generation sequencing experiments.

Here we have chosen to spike-in these uniquely tagged SASI fragments at a level of approximately 0.1% of the sample concentration. The presence of these fragments does not sacrifice significant sequence yield and sufficient numbers of identifiable SASI fragments allow cross-contamination to be readily detected. At this spike-in level sample identification and detection of sample mix-ups and cross-contaminations can be reliably detected down to 1% of the sample concentration. The tested conditions represent a subset of those that could be practically employed. The level of SASI fragment spike-in could be varied according to the desired cross-contamination detection sensitivity. With 96-plex sequencing on a single HiSeq 2000 lane, e.g., the majority of sample datasets had on the order of 1000 SASI reads. If one wanted to multiplex 384 samples instead and retain the same sensitivity, one would have to increase the SASI spike-in level 4-fold to 0.4%. Conversely, if one wanted to multiplex fewer samples, then the level of spike-in could be reduced. Increasing the sensitivity to detect cross-contamination ten-fold (to 0.1%), would require increasing the spike-in level ten-fold. Thus the use of SASI-Seq could be tailored to the requirements of the individual laboratory and possibly the requirements of individual experiments. Typical resequencing experiments involving at least 30-fold coverage will be largely tolerant of low levels of cross contamination but experiments designed to identify rare variants in a population, or low coverage sequencing experiments may be more sensitive. The ability to estimate cross contamination levels would then be valuable in reporting the level of background noise, enabling confidence levels to be ascribed to observations and analysis settings (e.g. required minimal coverage) to be modified. Knowledge of cross contamination levels could also be a valuable tool for operational monitoring and process optimization. Amongst the background levels of contamination present in the results presented here a 0.1% spike in level can cleanly report sample contamination levels down to 1%. This is significant since variation rarer than this is cannot be detected above the level of sequencing error, unless specific rare variant detection strategies are employed [35]. Since such strategies can theoretically detect 1 error in one billion they will be extremely sensitive to contamination and as such best performed in isolation, in which case SASI-seq would not be required.

Each laboratory may also want to consider the number of unique SASI fragments it employs. Ideally each and every sample would have a uniquely barcoded SASI fragment set, but when sequencing very large numbers of samples, that would be too expensive; each barcoded oligonucleotide has to be individually synthesised and SASI fragments need to be amplified, purified, quantified and quality controlled. Additionally, barcode sequences allowing such complexity and with an edit distance of 5, would get very long. Equally, utilising a set of just 96 SASI fragments is likely to be insufficient for a lab handling thousands of samples each month. We have shown that multiple SASI fragments can be added to a sample and that the presence of each can be detected following sequencing. Thus we envisage applying SASI-Seq in a combinatorial fashion so as to introduce complexity. For example, if two SASI fragments from a 96 uniquely barcoded set of SASI fragments were added to each sample there would be a maximal 4,560 degree of complexity ((96 × 95)/2), and if one had a 384 uniquely barcoded set of SASI fragments adding two SASI fragment sets per sample would give 73,536 possibilities. Though the application of SASI-Seq on a lower level could be tracked manually, its large-scale implementation requires LIMS tracking, registration of SASI barcodes to sample and post-sequencing analysis such that the appearance of the correct SASI barcode, along with any unexpected barcodes, can be reported.

## Conclusions

SASI-Seq is a simple, inexpensive and flexible tool that enables sample assurance, allows deconvolution of sample mix-ups and reports levels of cross-contamination between samples throughout NGS workflows. Its application will provide a guarantee that data generated originates from the intended sample and only the intended sample. This should increase the accuracy of epidemiological studies, increase the power of causal variant detection and give increased confidence to clinical conclusions taken from sequencing data.

## Methods

### Genomic DNA

Human DNA was purchased from Promega (Cat. no. G304A). Staphylococcus aureus TW20 genomic DNA was a gift from Jodi Lindsay, St George's Hospital Medical School, University of London.

### SASI fragment preparation

The three SASI amplicons were prepared by PCR using the following primers (obtained from IDT):

Forward A 214 bp fragment primers

{optional barcode sequence}GGCGCTCGTCTTTGGTATGTA

Forward B 397 bp fragment primers

{optional barcode sequence}TGAATTGTTCGCGTTTACCTT

Forward C 568 bp fragment primers

{optional barcode sequence}GTACGCTGGACTTTGTAGGAT

Reverse primer

{reverse complement of barcode sequence}GGCGTCCATCTCGAAG

Each amplification reaction comprised 1 ng of PhiX174 RFII DNA (NEB #N3022L), 200pM of appropriate forward primer, 200pM reverse primer and 1× Kapa HiFi mastermix (KK2602). All PCR was performed on an MJ Tetrad2 thermal cycler with the following conditions: 98°C for 2 minutes; 20 cycles of 98°C for 20 seconds, 55°C for 30 seconds, 72°C for 30 seconds; 72°C for 3 minutes. Amplicons were purified using a 1:1 ratio of AMPure XP beads (Beckman Coulter Inc.) and eluted in 30 μl of 10 mM Tris-HCl, pH8.5, prior to analysis on a Fragment Analyzer (Advanced Analytical) and fluorimetric quantification using Quant-iT™ PicoGreen ® dsDNA Reagent and DNA standards of known concentration. Each set of three SASI fragments bearing the same barcode was mixed in equimolar amounts and adjusted to a final concentration of 0.5 ng/μl. Barcoded SASI fragments were added to samples at a dosing of 0.1% which we considered would give sufficient numbers of SASI reads to detect low level cross-contamination without significantly sacrificing data yield.

### Illumina library construction

DNA (0.5 μg in 100 μl of 10 mM Tris-HCl, pH8.5) was sheared in an AFA microtube using a Covaris S2 device (Covaris Inc.), with the following settings: for 200 bp fragments (duty cycle 20, intensity 5, 200 cycles/burst, 90 sec), for 300 bp fragments (duty cycle 20, intensity 5, 200 cycles/burst, 45 sec), for 400 bp fragments (duty cycle 20, intensity 5, 200 cycles/burst, 30 sec) and for 500 bp fragments (duty cycle 5, intensity 3, 200 cycles/burst, 80 sec).

Sheared DNA was purified by binding to an equal volume of AMPure XP beads (Beckman Coulter, Inc.) and eluted in 32 μl of 10 mM Tris-HCl, pH8.5. End-repair, A-tailing and paired-end adapter ligation were performed using NEBNext reagents from New England Biolabs, with purification using a 1:1 ratio of AMPure XP beads to sample between each reaction. After ligation, excess adapters and adapter dimers were removed using two Ampure XP clean-ups, first with a 1.1:1 ratio of standard AMPure XP beads to sample, followed by a 0.7:1 ratio. Adapter-ligated fragments were amplified using Kapa HiFi polymerase (Kapa Biosystems cat. no. KK2602) as previously described [30] with 200 nM final concentration of primer PE1.0 and modified multiplexing PE2.0 primers.

After PCR, excess primers and any primer dimer were removed using two AMPure XP clean-ups, with a 0.7:1 ratio of AMPure XP beads. All libraries were quantified by real-time PCR, using the SYBR Fast Illumina Library Quantification Kit (Kapa Biosystems cat. no. KK4834), prior to pooling and sequencing.

Libraries in 96-well microtitre plates were prepared as above, with the following modifications. DNA was sheared to a mean fragment size of 300 bp in Covaris 96 microTUBE plates (Covaris Inc., part no. 520078) using a Covaris E210 instrument with the settings: duty cycle 10, intensity 5, 200 cycles/burst, 60 sec. PrePCR processing was performed using a Beckman FxP dual arm liquid handling platform. A Caliper Zephyr liquid handler was used for a single post-PCR cleanup, using a 0.7:1 ratio of AMPure XP beads to DNA.

### Agarose-gel size selection

DNA (30 μl) was mixed with 6 μl of 6× loading dye (Qiagen) and loaded into a well of a 2% low-range Ultra-agarose gel (BioRad 161-3107) set within an EM100 10 × 10 cm horizontal electrophoresis chamber (Engineering and design plastics Ltd.) using TAE running buffer [3]. The sample was run alongside a 10 μl aliquot of low molecular weight DNA ladder (NEB, N3233L) at 6 V/cm for 2 hours, after which time the gel was stained for 30 minutes in 50 ml of TAE buffer containing SYBR green (Invitrogen) at 1/10,000 dilution. The gel was visualized on a Dark Reader (Clare Chemical Research Inc.) and gel slices containing DNA fragments of the desired size ranges were excised with a scalpel. DNA was extracted from gel slices using the Qiaquick Gel Extraction Kit (Qiagen), with dissolution of the gel slice in QG buffer at room temperature for 10 minutes* and eluted in 30 μl of 10 mM Tris-HCl, pH8.5.

* We have noted previously [26] that use of the standard Qiagen gel-extraction protocol with dissolution of the gel slice at 55°C can result in the loss of very AT-rich sequences.

### AMPure XP size selection

AMPure XP SPRI beads (Beckman Coulter Inc.) were used according to the manufacturer’s protocol, with binding for 5 minutes, two washes with 80% ethanol, air drying for 10 minutes and elution into 30 μl of 10 mM Tris-HCl, pH8.5. Unless stated otherwise, a 1:1 ratio of beads to sample was used, resulting in removal of most <200 bp fragments.

To select 400-600 bp fragments, DNA was double size selected using AMPure beads by first adding a 0.6:1 (beads:sample) aliquot of beads to bind >600 bp material. The supernatant was removed from these beads and transferred to a fresh tube. To this supernatant an extra 0.12:1 aliquot of beads was then added to bind fragments >400 bp. These beads were washed, dried and DNA (containing fragments of mostly 400 to 600 bp) eluted from them into 30 μl of 10 mM Tris-HCl, pH8.5.

### Pippin prep size selection

DNA (30 μl) was mixed with 10 μl loading solution and loaded into the well of a 2%-agarose cassette (Sage Science; CSD2010 with ethidium bromide, or CEF2010 dye free) and run according to the manufacturer’s instructions. DNA was collected from the elution port and purified using the Qiaquick PCR Purification Kit (Qiagen), prior to further use, to remove ethidium bromide. We found that the Pippin Prep elution buffer is alkaline and so eluted DNA does not bind well to Qiagen columns. To overcome this issue, we routinely add 1 μl of 3 M sodium acetate, pH5.2 to the eluate before adding the Qiagen binding buffer.

### Caliper labchip XT size selection

DNA (10 μl) was mixed with 2 μl of loading solution and loaded into the sample well of a DNA 750 chip (part no. 760541) and run according to the manufacturer’s instructions.

### SureSelect target enrichment

Whole exome targeted enrichment libraries were prepared using the Agilent SureSelect XT Targeted Enrichment system according to manufacturer’s protocols, except that the adapters and PCR indexing primers outlined in the Illumina Library Construction section above were used, along with blocking oligos complementary to the indexing primers containing universal deoxyinosine bases over the index sequence.

Where we sought to actively enrich SASI fragments, we included a single-stranded DNA probe complementary to the region of the phiX genome present in all SASI fragments into the SureSelect Human All Exon 50 Mb (ELID S02972011) v5 bait mix. Specifically this oligo: /5’Biotin/CGGCGTCCATCTCGAAGGAGTCGCCAGCGATAACCGGAGTAGTTGAAATGGTAATAAGACGACCAATCTGACCAGCAAGGAAGCCAAGATGGGAAAGGTCATGCGGCATACGCTCGGCG was synthesised as a 4nmole scale ultramer from IDT and was included in the hybridisation in a range of final concentration of 10 μM.

### Illumina sequencing

Most samples were sequenced on an Illumina MiSeq instrument with the appropriate length index read and v2 chemistry. Runs were either 75, 100 or 130 base paired end, though 50 base single-end sequencing was used for barcode frequency determination. For the 96-sample experiments, 75 base paired-end sequencing was performed on an Illumina HiSeq.

To eliminate the possibility of any potential sample carryover between MiSeq runs that may affect observed background levels of barcode sequences, we scheduled runs such that samples with different barcoding strategies, or no barcoding, were alternated with runs employing the barcoding schema here described.

### Data processing

After sequencing, reads were mapped to each genome reference sequence using SMALT [36]. SAMtools [37] was then used to generate pileup and coverage information from the mapping output. SMALT was also used for variant analysis.

### Availability of supporting data

All datasets have been deposited in the ENA read archive under accession number ERP001281. A list of the experimental conditions pertaining to each sample submission is given in s sample accession numbers please see Additional file 8: Table S7.

## Electronic supplementary material

Additional file 1: Table S1: Sequences of Illumina 9-mer barcodes and corresponding PCR indexing oligos. (XLS 146 KB)

Additional file 2: Figure S1: Post sequencing mapped insert size distributions for a random 10,000 read subset, following tight Pippin Prep size selection. (PPTX 84 KB)

Additional file 3: Table S2: Results of oligo purity experiment comparing HPLC, PAGE and TruGrade processed indexing oligos. In each set of libraries, some barcoded libraries have been deliberately omitted, in order to assess the level of background contamination of those barcodes. (XLS 32 KB)

Additional file 4: Table S3: Demonstration of barcode cross-contamination through sequence drift due to errors in sequencing. (XLS 108 KB)

Additional file 5: Table S4: Sequences of Illumina 11-mer barcodes and corresponding PCR indexing oligos. (XLS 110 KB)

Additional file 6: Table S5: Frequency of matches to each of the 384 Illumina 11-mer barcode sequences from sequencing runs of odd and even number barcoded libraries. (XLS 84 KB)

Additional file 7: Table S6: Demonstration of the utility of SASI-Seq for sample assurance. Matrix of library barcode versus expected spike-in barcode for that library for the 96-sample SASI-Seq experiment. (XLS 88 KB)

Additional file 8: Table S7: List of sequencing datasets used in this study and submitted to ENA read archive under accession number ERP001281. (XLS 98 KB)

## Abbreviations

NGS: Next-generation sequencing
Tm: Melting temperature
bp: Base pairs
PCR: Polymerase chain reaction
HPLC: High performance liquid chromatography
PAGE: Polyacrylamide gel electrophoresis.
