# Supplementary figures and images for: SASI-Seq: sample assurance Spike-Ins, and highly differentiating 384 barcoding for Illumina sequencing
Source: BMC Genomics. 2014 Feb 7;15(1):110. doi: 10.1186/1471-2164-15-110 (PMC4008303; doi:10.1186/1471-2164-15-110)

## Slide 1
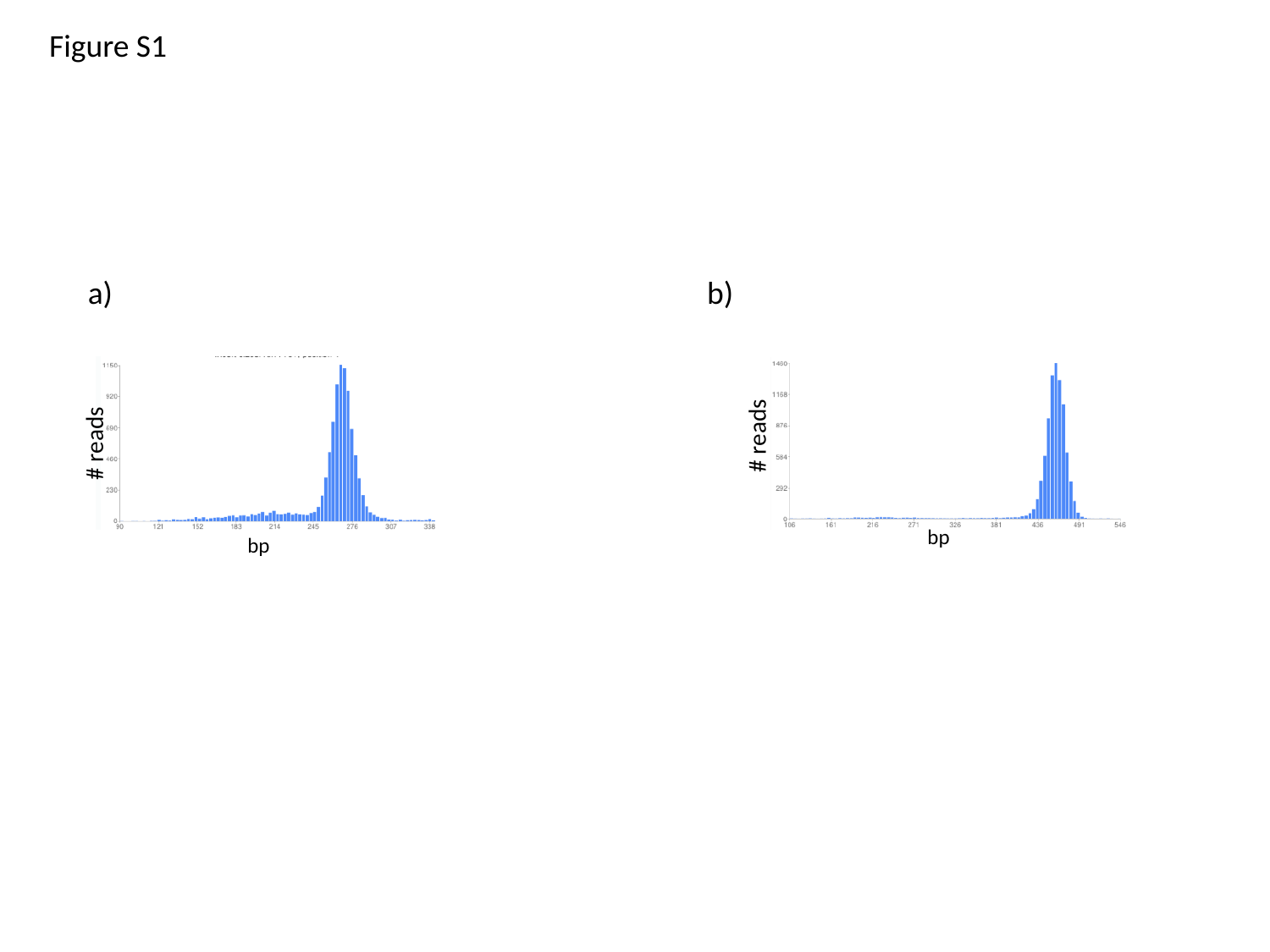

Figure S1
a)
b)
# reads
# reads
bp
bp

Supplement: Supplementary file 2 — Additional file 2: Figure S1: Post sequencing mapped insert size distributions for a random 10,000 read subset, following tight Pippin Prep size selection. (PPTX 84 KB) [file 12864_2013_7011_MOESM2_ESM.pptx]
